# Supplementary material for: Comparative study of protease hydrolysis reaction demonstrating Normalized Peptide Bond Cleavage Frequency and Protease Substrate Broadness Index
Source: PLoS One. 2020 Sep 21;15(9):e0239080. doi: 10.1371/journal.pone.0239080 (PMC7505449; doi:10.1371/journal.pone.0239080)
Supplement: Attachment — Submitted filename: Response to Reviewers-20200827.docx [file pone.0239080.s004.docx]

To Dr Luis Menéndez-Arias

From Dr Shukun Yu and coauthors.

Aarhus Denmark Aug. 27, 2020.

Dear Dr Luis Menéndez-Arias,

Thank you for your email dated on Aug 26, 2020 on our manuscript PONE-D-20-14957R1. We are very glad that all the three reviewers are happy with our revisions. Reviewer 1 has no further comments, Reviewer 3 wanted to correct the typo of trptophan to tryptophan (done). Reviewer 2 has some suggestions on the wording and spelling. All of them are accepted and corrected (see below). Thank you and all the three reviewers for their very good job done.

*Reviewer #2: The authors have made the changes suggested by myself and the other reviewers and the paper is not acceptable for publication provided the following minor points, mostly errors in grammar introduced during the revision, are addressed.*

Line 428: Remove "that" from "This is because that a protein".**_Done**
Line 429: It would be better to say that "each peptide bond can be regarded as a potential substrate".**_Done**
Line 444: insert "out" after "carried".**_Done**
Line 483: It is usual for numbers under ten to be spelt out in full; so replace "4" with "four". **_Done**
Line 486: replace "presented" with "represented".**_Done**
Line 487-488: There is no need to capitalize "succinyl". Succinyl-AAPX-pNA is not a single substrate but range of substrates depending on the amino acid in the P1 position (shown as "X"), so replace "has" with "have" and "is" with "are". I suggest putting commas around the clause "which has been used ... at P1".**_Done**
Line 497: "triplicate" should be plural. **_Done**
Line 499: Replace "Beside" with "Besides".**_Done**
Line 512: replace "present" with "represent".**_Done**
lines 513-515: Considering that a prerequisite of the experimental procedure is the denaturation of SPI with urea prior to digestion, it seems unlikely that much tertiary structure of disulfide bridges remain. So this negates one of the advantages of SPI over the oxidized insulin B-chain. **_the word tertiary is deleted.**

We also made minor improvements of Fig 1 (added 20:0.5:0.5, 100:0.5:0.5), Fig 3A (deleted a space), Fig 8A and 8B (corrected typos). All these figures were saved as TIF and converted at <https://pacev2.apexcovantage.com>

**Bst rgs**, Dr Shukun Yu, Senior staff scientist,  **DuPont Nutrition & Biosciences,** Edwin Rahrs Vej 38, DK 8220 Brabrand,  Aarhus, Denmark, Tel: 0045 8943 5495; Cell: +45 24675 165. External Professor at **Lund University Sweden** (2008-14). Pls watch our 1 min video on phytase: <https://youtu.be/hvQK6XNd9Z0>

**PS, the letter from Dr Luis Menéndez-Arias dated on Aug. 26, 2020**

**From:** em.pone.0.6d8ed4.00c6963e@editorialmanager.com <em.pone.0.6d8ed4.00c6963e@editorialmanager.com> **On Behalf Of** PLOS ONE
**Sent:** 26. august 2020 17:58
**To:** Shukun Yu <Shukun.Yu@dupont.com>
**Subject:** [EXTERNAL] PLOS ONE Decision: Revision required [PONE-D-20-14957R1] - [EMID:f868c8eaeaef3404]

PONE-D-20-14957R1
Comparative study of protease hydrolysis reaction demonstrating Normalized Peptide Bond Cleavage Frequency and Protease Substrate Broadness Index
PLOS ONE

Dear Dr. Yu,

Thank you for submitting your revised manuscript to PLoS ONE. After this round of revision, reviewers think that the manuscript has been improved and most queries and questions have been satisfactorily addressed. However, there are still some minor corrections to be done before the manuscript is accepted for publication (see reports below).

Although your manuscript needs revision, we believe that this is minor and the article should be publishable after appropriate changes have been done. If you are prepared to undertake the work required, I would be pleased to reconsider my decision.

Please submit your revised manuscript by Oct 10 2020 11:59PM. If you will need more time than this to complete your revisions, please reply to this message or contact the journal office at [plosone@plos.org](mailto:plosone@plos.org). When you're ready to submit your revision, log on to [https://www.editorialmanager.com/pone/](https://urldefense.proofpoint.com/v2/url?u=https-3A__www.editorialmanager.com_pone_&d=DwQGaQ&c=zRqMG_fghhK--2M6Q5UUdA&r=8lt1N0nDu8C-Qut3wIs8uswb4qcT768us1nJVr_HwDs&m=pbzCBqDr0XBe2X8dutDJXL8Nd0hGtanjb6wrctZUZ_U&s=AtxrElIKzfdWIvTehyPq55hjcFcyDr9IidwS9k6sync&e=) and select the 'Submissions Needing Revision' folder to locate your manuscript file.

Please include the following items when submitting your revised manuscript:

- A rebuttal letter that responds to each point raised by the academic editor and reviewer(s). You should upload this letter as a separate file labeled 'Response to Reviewers'.
- A marked-up copy of your manuscript that highlights changes made to the original version. You should upload this as a separate file labeled 'Revised Manuscript with Track Changes'.
- An unmarked version of your revised paper without tracked changes. You should upload this as a separate file labeled 'Manuscript'.

If you would like to make changes to your financial disclosure, please include your updated statement in your cover letter. Guidelines for resubmitting your figure files are available below the reviewer comments at the end of this letter.

If applicable, we recommend that you deposit your laboratory protocols in protocols.io to enhance the reproducibility of your results. Protocols.io assigns your protocol its own identifier (DOI) so that it can be cited independently in the future. For instructions see: [http://journals.plos.org/plosone/s/submission-guidelines#loc-laboratory-protocols](https://urldefense.proofpoint.com/v2/url?u=http-3A__journals.plos.org_plosone_s_submission-2Dguidelines-23loc-2Dlaboratory-2Dprotocols&d=DwMGaQ&c=zRqMG_fghhK--2M6Q5UUdA&r=8lt1N0nDu8C-Qut3wIs8uswb4qcT768us1nJVr_HwDs&m=pbzCBqDr0XBe2X8dutDJXL8Nd0hGtanjb6wrctZUZ_U&s=fB1atHxlmzbFLRsC9ioj_p8A7zVnWYAeBFoVYdx6DW4&e=)

We look forward to receiving your revised manuscript.

Kind regards,

Luis Menéndez-Arias, Ph. D.
Academic Editor
PLOS ONE


[Note: HTML markup is below. Please do not edit.]

Reviewers' comments:

Reviewer's Responses to Questions

**Comments to the Author**

1. If the authors have adequately addressed your comments raised in a previous round of review and you feel that this manuscript is now acceptable for publication, you may indicate that here to bypass the “Comments to the Author” section, enter your conflict of interest statement in the “Confidential to Editor” section, and submit your "Accept" recommendation.

Reviewer #1: All comments have been addressed

Reviewer #2: All comments have been addressed

Reviewer #3: All comments have been addressed

2. Is the manuscript technically sound, and do the data support the conclusions?

The manuscript must describe a technically sound piece of scientific research with data that supports the conclusions. Experiments must have been conducted rigorously, with appropriate controls, replication, and sample sizes. The conclusions must be drawn appropriately based on the data presented.

Reviewer #1: Yes

Reviewer #2: Yes

Reviewer #3: Yes

3. Has the statistical analysis been performed appropriately and rigorously?

Reviewer #1: Yes

Reviewer #2: Yes

Reviewer #3: Yes

4. Have the authors made all data underlying the findings in their manuscript fully available?

The [PLOS Data policy](https://urldefense.proofpoint.com/v2/url?u=http-3A__www.plosone.org_static_policies.action-23sharing&d=DwMGaQ&c=zRqMG_fghhK--2M6Q5UUdA&r=8lt1N0nDu8C-Qut3wIs8uswb4qcT768us1nJVr_HwDs&m=pbzCBqDr0XBe2X8dutDJXL8Nd0hGtanjb6wrctZUZ_U&s=A1whsccIlQObQiYgadPayd-iDZ3U5Z6H2agkmfhReMk&e=) requires authors to make all data underlying the findings described in their manuscript fully available without restriction, with rare exception (please refer to the Data Availability Statement in the manuscript PDF file). The data should be provided as part of the manuscript or its supporting information, or deposited to a public repository. For example, in addition to summary statistics, the data points behind means, medians and variance measures should be available. If there are restrictions on publicly sharing data—e.g. participant privacy or use of data from a third party—those must be specified.

Reviewer #1: Yes

Reviewer #2: Yes

Reviewer #3: Yes

5. Is the manuscript presented in an intelligible fashion and written in standard English?

PLOS ONE does not copyedit accepted manuscripts, so the language in submitted articles must be clear, correct, and unambiguous. Any typographical or grammatical errors should be corrected at revision, so please note any specific errors here.

Reviewer #1: Yes

Reviewer #2: Yes

Reviewer #3: Yes

6. Review Comments to the Author

Please use the space provided to explain your answers to the questions above. You may also include additional comments for the author, including concerns about dual publication, research ethics, or publication ethics. (Please upload your review as an attachment if it exceeds 20,000 characters)

Reviewer #1: (No Response)

Reviewer #2: The authors have made the changes suggested by myself and the other reviewers and the paper is not acceptable for publication provided the following minor points, mostly errors in grammar introduced during the revision, are addressed.

Line 428: Remove "that" from "This is because that a protein".
Line 429: It would be better to say that "each peptide bond can be regarded as a potential substrate".
Line 444: insert "out" after "carried".
Line 483: It is usual for numbers under ten to be spelt out in full; so replace "4" with "four".
Line 486: replace "presented" with "represented".
Line 487-488: There is no need to capitalize "succinyl". Succinyl-AAPX-pNA is not a single substrate but range of substrates depending on the amino acid in the P1 position (shown as "X"), so replace "has" with "have" and "is" with "are". I suggest putting commas around the clause "which has been used ... at P1".
Line 497: "triplicate" should be plural.
Line 499: Replace "Beside" with "Besides".
Line 512: replace "present" with "represent".
lines 513-515: Considering that a prerequisite of the experimental procedure is the denaturation of SPI with urea prior to digestion, it seems unlikely that much tertiary structure of disulfide bridges remain. So this negates one of the advantages of SPI over the oxidized insulin B-chain.

Table 3. "Tryptophan" is mis-spelt.

Reviewer #3: The authors Yu, Thoergersen and Kragh have done a very good job in revising their manuscript. I think no further explanation of my answers to the above questions is required. As I read all their responses to all three reviewers comments, it is clear that they meticulously corrected every critical point. Thus, I can only suggest immediate acception of the study and would like to congratulate the authors for their work. It would be interesting to see, whether they can further develop the two novel concepts of NPBCF and PSBI beyond the food protein context. In fact, I see some potential for other applications in the protease field.

7. PLOS authors have the option to publish the peer review history of their article ([what does this mean?](https://urldefense.proofpoint.com/v2/url?u=https-3A__journals.plos.org_plosone_s_editorial-2Dand-2Dpeer-2Dreview-2Dprocess-23loc-2Dpeer-2Dreview-2Dhistory&d=DwMGaQ&c=zRqMG_fghhK--2M6Q5UUdA&r=8lt1N0nDu8C-Qut3wIs8uswb4qcT768us1nJVr_HwDs&m=pbzCBqDr0XBe2X8dutDJXL8Nd0hGtanjb6wrctZUZ_U&s=Ee9gFtt7mMAztYCu744wY3TdfWSKDZgBP-IvEyU7hxw&e=)). If published, this will include your full peer review and any attached files.


If you choose “no”, your identity will remain anonymous but your review may still be made public.


**Do you want your identity to be public for this peer review?** For information about this choice, including consent withdrawal, please see our [Privacy Policy](https://urldefense.proofpoint.com/v2/url?u=https-3A__www.plos.org_privacy-2Dpolicy&d=DwMGaQ&c=zRqMG_fghhK--2M6Q5UUdA&r=8lt1N0nDu8C-Qut3wIs8uswb4qcT768us1nJVr_HwDs&m=pbzCBqDr0XBe2X8dutDJXL8Nd0hGtanjb6wrctZUZ_U&s=OG-ChBC-6NiT-C0HjZ3sgBfsaATw3FEeaDiIwSQeFvo&e=).

Reviewer #1: No

Reviewer #2: No

Reviewer #3: No

[NOTE: If reviewer comments were submitted as an attachment file, they will be attached to this email and accessible via the submission site. Please log into your account, locate the manuscript record, and check for the action link "View Attachments". If this link does not appear, there are no attachment files.]

While revising your submission, please upload your figure files to the Preflight Analysis and Conversion Engine (PACE) digital diagnostic tool, [https://pacev2.apexcovantage.com/](https://urldefense.proofpoint.com/v2/url?u=https-3A__pacev2.apexcovantage.com_&d=DwMGaQ&c=zRqMG_fghhK--2M6Q5UUdA&r=8lt1N0nDu8C-Qut3wIs8uswb4qcT768us1nJVr_HwDs&m=pbzCBqDr0XBe2X8dutDJXL8Nd0hGtanjb6wrctZUZ_U&s=aHMoh--ua3EvgAhH0p2DhRHbjjVWu6_t1rUGesUxmtk&e=). PACE helps ensure that figures meet PLOS requirements. To use PACE, you must first register as a user. Registration is free. Then, login and navigate to the UPLOAD tab, where you will find detailed instructions on how to use the tool. If you encounter any issues or have any questions when using PACE, please email PLOS at [figures@plos.org](mailto:figures@plos.org). Please note that Supporting Information files do not need this step.
